# Supplemental online resources improve data literacy education: Evidence from a social science methods course

**DOI:** 10.1371/journal.pone.0315318

**Published:** 2024-12-19

**Authors:** Marco Alcocer, Leonardo Falabella, Alexandra Lange, Nicholas Smith, Maureen Feeley

**Affiliations:** 1 Department of Political Science, University of California, San Diego, La Jolla, California, United States of Ameirca; 2 The Center for Peace and Security Studies, University of California, San Diego, La Jolla, California, United States of Ameirca; Beijing University of Technology, CHINA

## Abstract

Despite the importance of data literacy skills for academic and professional careers, learning these skills is a source of stress and difficulty for undergraduate students. This study first introduces an online supplemental instruction resource to support student learning in an introductory data analysis course at a large public university. To evaluate its impact, we conduct a pre-registered double-blind within-subject experiment. Each student is randomly assigned to a subset of the online supplemental instruction modules and takes an exam assessing concepts covered by the course material and supplemented by the modules. Access to the online supplemental instruction modules improves student performance on exam questions, and students who engage with the modules improve exam scores even more. We find no differential impacts based on pre-treatment GPA or underrepresented status, and a post-experiment survey suggests that the online supplemental instruction modules are also well-received by students. This study shows that asynchronous online supplemental instruction resources are a promising way to support student learning in data literacy.

## Introduction

Data literacy skills are foundational for academic and professional success and ever more necessary for students’ everyday lives [1]. Addressing the challenges individuals and communities face—locally, nationally, and globally—increasingly requires thinking critically about empirical evidence. Learning these skills, however, is a source of stress and difficulty for students across disciplines, particularly for those with high levels of mathematics anxiety [2–24]. To fulfill the public mission of preparing citizens to address 21st-century problems, higher education institutions must respond to these challenges by supporting student learning in data literacy.

In response, some universities have developed online supplemental instruction resources (OSI) that complement course instruction during the academic term. Many universities have also developed in-person supplemental instruction and scholars have found these to be largely successful [25, 26]. Yet, we know little about the effects of OSI on student learning. Existing studies remain limited by methodological challenges, including small sample sizes [27], selection bias [28, 29], or comparing in-person SI to OSI without a control group [30–32]. Furthermore, to our knowledge, OSI interventions on data literacy have yet to be tested empirically.

In this study, we introduce an OSI resource and show that it substantially improves data literacy education. This study was pre-registered before collecting outcome data at the EGAP Registry, accessible at https://osf.io/scx6r. This study was conducted with the approval of UCSD’s Institutional Review Board, protocol #170886, and a written form of consent was obtained from all participants. The OSI resource is a pre-built and self-contained series of interactive modules with videos, quizzes, and summaries of key concepts. To evaluate its impact, we conducted a double-blind, within-subject experiment in a social science course at a large public university. The OSI resource is publicly available at the American Political Science Association (APSA) website. It can accessed at https://educate.apsanet.org/home-foundations-of-quantitative-research-in-political-science-org.

We made the OSI modules available five weeks before an exam, with each student receiving a random subset of the modules. Each module supplemented specific exam questions. The exam is available in this paper’s Supplemental Information (SI) section. It contained short-answer questions assessing students’ understanding of key concepts and ability to apply them to real-world examples. We then compare the exam scores for questions that were supplemented with OSI modules with those that were not, accounting for variations in student ability and question difficulty.

We find that the OSI modules significantly improved student performance on exam questions, with larger effects for students who engaged more deeply with the resources. Specifically, giving students access to the modules increased exam question grades by 3.8 percentage points. Students who viewed the modules increased their scores by 5.3 percentage points, and those who answered a module quiz increased their scores by nearly 11 percentage points. Interestingly, we find no evidence that the treatment had statistically significant differential effects on students based on their pre-treatment cumulative GPA or underrepresented status. Finally, a voluntary post-experiment survey finds that an overwhelming majority of respondents believe the modules helped them learn and achieve higher grades and that they would recommend the modules to other students. Our results demonstrate the effectiveness of OSI as a tool for improving data literacy in higher education and provide suggestive evidence that they are well-received by students.

In addition to the OSI modules and the experimental results, our research design is also an important contribution. First, it provides a more ethical way of testing the effects of OSI because it ensures that all students have access to OSI modules. Second, because each student functions as a block within which modules are randomized, this design allows for the inclusion of student fixed effects that absorb student-specific variance. Finally, the within-subject design generates more observations than traditional designs, increasing statistical power. This may be especially useful for educational researchers designing experiments constrained by small class sizes.

As demand for university graduates with proficiency in data literacy increases, asynchronous OSI tools may offer a time- and cost-efficient way to support student learning. The resource introduced here is pre-built and self-contained, requiring minimal instructional support after its implementation. Modules can be accessed by students at any time, and in any order, as needed, and interactive quizzes are self-graded.

## Data literacy education and course background

We follow the literature in understanding data literacy as “the ability to ask and answer real-world questions from large and small data sets through an inquiry process” [33]. Data literacy is based on core skills, such as collecting, manipulating, and interpreting data, developing hypotheses, and transforming information into actionable knowledge [33–36].

Research shows that students in the social sciences struggle in data literacy courses for various reasons. First, students often arrive with varying levels of prior exposure to prerequisite mathematical concepts as well as “fixed mindsets” or preconceptions about their ability to succeed [15, 16]. Second, the type of learning required in data-related courses is often different from other courses because learning data skills tends to be more linear and cumulative [15, pg. 527]. Third, students often mistakenly assume they can succeed in these classes by memorizing statistical formulae, rather than focusing their efforts on understanding underlying principles and logic [15]. And fourth, many of these courses have high student-to-faculty and student-to-TA ratios, which can constrain instructors’ and TAs’ ability to provide the frequent, individualized feedback that students need [37].

At UC San Diego, the Department of Political Science requires that all of its undergraduate majors take a 10-week introductory course on research methods and data literacy, Political Inquiry (POLI 30). POLI 30 introduces students to the fundamentals of data analysis, including instruction in statistical software, probability theory, measurement, inference, research design, hypothesis testing, linear regression, and other basic quantitative methods. These skills are a fundamental part of data literacy, especially with respect to research methods, but do not address other aspects of data literacy such as data privacy and ownership, data-driven processes, or predictive modeling.

The course is offered every quarter (Fall, Winter, Spring, Summer) and typically enrolls between 200 and 250 students each quarter during the academic year (Fall-Spring). The course is taught by multiple instructors, resulting in some variation in course content, but core concepts and learning outcomes remain consistent across instructors. In each offering of the course during the academic year, students meet twice a week for 50-minute lectures led by the course instructor, with an additional weekly meeting (50 minutes) in smaller discussion sections (approximately 30 students per section) led by graduate student TAs.

POLI 30 has double the average DFW rate (D grade, fail, or withdraw) of all courses in the Political Science curriculum. This is perhaps not surprising, given extant research on how and why social science students struggle in data courses. The authors of this study have witnessed this firsthand while teaching POLI 30. Many students who enroll in the course exhibit low confidence in their ability to learn its content, and demonstrate high levels of stress and anxiety. Given the fast-paced nature of the 10-week quarter system, students typically struggle to keep up with the content, an issue exacerbated by the cumulative nature of learning in the course where mastery of new knowledge and skills is contingent on mastery of earlier material. The challenges faced by students in learning such core skills have motivated us to develop supplemental material to support their learning.

## Foundations of data analysis for social science

To address the challenges of data literacy education, we created an asynchronous OSI resource, “Foundations of Data Analysis for Social Science.” The OSI resource contains ten modules, four of which are tested in our experiment. The modules focus on foundational data literacy topics students have historically struggled with: (1) Introduction, (2) Research Questions, Theories, and Hypotheses, (3) Introduction to Variables, (4) Confounding and Intervening Variables, (5) Research Design, (6) Introduction to Inference, (7) Hypothesis Testing, (8) Regression Analysis, (9) Working with Data, and (10) Visualizing Data. See S1 File for details on the content of each module. Modules 3–6 are tested in our experiment, with each student obtaining access to a random subset of two modules from the start of the course until the midterm exam. Modules 1 and 2 were accessible to all students since the course started, and modules 7–10 were not taught during the first half of the course when we implemented the experiment. After the midterm exam, all students were given access to all ten modules. This constrained our ability to test the efficacy of additional modules but was necessary so that students had full support for the final exam.

**Fig 1:**
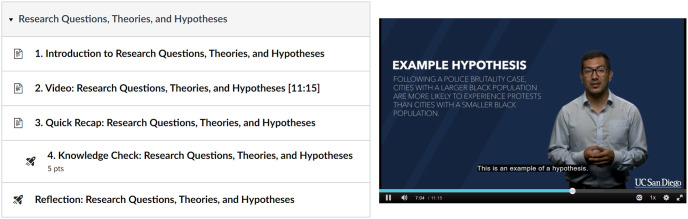
User interface of OSI modules as seen on Canvas. Left screenshot is from the Canvas menu showing the Research Questions, Theories, and Hypotheses module. Right screenshot is from the Research Questions, Theories, and Hypotheses video.

Each of the content-based modules includes: (1) a brief textual overview of the module’s content and learning outcomes; (2) one or more short videos (approximately seven to ten minutes) that embed concepts within a motivating social or political problem, focus on addressing misconceptions, and explain the logic underlying concepts; (3) a brief textual summary or “recap” of the module with links to additional supplementary materials for students to “dig deeper,” (4) a “knowledge check”—interactive quiz(zes) with feedback to assess how well students understood the module, and (5) a “reflection” opportunity that invites students to identify what they have learned and provide feedback on the module. Fig 1 displays the user interface of one of the modules and a screenshot from an instructional video.

All modules begin with a clear statement of learning outcomes, which aligns with research on effective pedagogy [38, 39]. Following previous research on teaching data literacy [15, 16] and effective pedagogy [38, 40], the modules are designed to introduce fundamental concepts in an intuitive way, rather than a more math-heavy or highly technical approach. The modules introduce real-world problems to motivate the logic underlying key concepts, address common misconceptions, and provide examples that highlight the potential applications of key concepts.

Building on research from cognitive science [40] and multimedia learning [41], as well as guidance from educational and technology specialists at UCSD, videos were kept relatively short (approximately seven to ten minutes) and were filmed in UCSD’s professional studio. Each video is accompanied by a “quick recap” that briefly summarizes the main learning objectives of the video as recommended by prior research in this area [27, 37, 38, 40, 42]. Module summaries are then followed by “knowledge checks” (i.e., quizzes) that provide students with opportunities to check their mastery of the module’s concepts. Knowledge check questions were carefully designed to probe common misconceptions and question banks were created to enable students to take multiple self-tests with immediate feedback on their level of mastery. The pedagogical value of providing students self-testing opportunities with prompt formative feedback is well established in the literature on human cognition [37, 38, 40, 42], but as discussed above, is often challenging to implement in large-enrollment courses such as POLI 30. The final section of each module asks students to reflect on their learning and to provide feedback on the module itself—both of which are pedagogical practices that research has demonstrated deepen learning, improve retention, and build metacognitive skills [38, 40]. Questions include: (1) “What are your main takeaways from this module?” (2) “What questions remain for you?” (3) “Do you have any feedback about this module to share? How can this module be improved?” and (4) “Please rate your agreement with the following statements,” with statements including (a) “I found this module helpful,” (b) “The video(s) in this module improved my understanding of this module’s content,” and (c) “The knowledge check(s) in this module improved my understanding of this module’s content.” Questions 3 and 4 were added after we conducted our experiment, but we include them here as a recommendation for obtaining low-effort student feedback, as students may not want to spend the time to answer open-ended questions.

Beyond these common elements of each module, the modules themselves are generally ordered from more foundational to more complex concepts. Depending on their needs, students are free to engage with modules in whichever order they choose. For ease of use, we chose to host the modules on UCSD’s central learning management system (LMS), Canvas. Canvas is an online learning management system (LMS) developed by Instructure and used by universities, educators, and students to manage and access course content, and is the default LMS at UCSD. This has the additional benefit of being able to share the resources broadly beyond UCSD using the “Canvas Commons” platform. At the time of writing this paper, the modules are available to all students in the University of California system via Canvas Commons. In typical offerings of POLI 30, students have access to all ten modules on a single Canvas course via a “join” link during the first week of the quarter. Once students join the OSI Canvas “course”, the modules populate their home Canvas page as a separate course. In our study, students received weekly email reminders to access the OSI resources to support their learning and help them prepare for exams. S1 File shows descriptive statistics about student use of the modules. We find that that students were generally motivated to view OSI pages but not as willing to answer quiz questions.

While the modules were designed by the authors, four of whom have been section leaders for POLI 30, we sought additional feedback to ensure the validity of the module content. First, we had four faculty members who regularly teach POLI 30 review the materials. We also received support from education specialists at the UCSD Teaching and Learning Commons and Education Technology Services. Finally, to improve accessibility and ease of use, we recruited five undergraduate research apprentices (RAs) who had recently taken POLI 30 to provide feedback and suggestions to ensure that the language in the modules was accessible and engaging for undergraduate students.

## Materials and methods

To evaluate the impact of the modules on student learning, we conducted a pre-registered double-blind experiment using a within-subject design. First, we designed a midterm exam where each question evaluated student knowledge on course material that was supplemented by a corresponding OSI module. This ensured that no student was disadvantaged since the midterm questions could be answered with just course material. We then randomly assigned each student to a subset of the OSI modules by giving them access to a Canvas page that only included the modules they were assigned. This means that students only received the treatment for some exam questions and not others. Thus, for any given student, an untreated exam question tested their knowledge on a topic that was presented in standard course material (textbook, lectures, discussion sections, and office hours), but for which they did not have access to the relevant OSI module. By contrast, a treated question tested a student’s knowledge on a topic presented in both standard course material and the corresponding OSI module. This design assured that each student had the opportunity to engage with two out of four supplementary modules.

Our sample consists of undergraduate students at UCSD who were enrolled in the course during Spring term of 2021, excluding those who opted out or did not take the midterm exam. All students who consented to participating in the experiment were assigned to treatment, including students who enrolled late. For the purposes of the experiment, we created six different Canvas courses, one for each treatment group. Students enrolled in POLI 30 during the Spring quarter of 2021 were randomly assigned to one of these six treatment groups. Students were introduced to the study and the opt-out IRB form during week 1 of the quarter.

Two modules were available for every treatment group: the module that introduced the resources and a module on research questions, theories, and hypotheses (these contain foundational knowledge and are therefore crucial to understanding subsequent modules). Each treatment group was also provided access to two additional modules randomly selected from a set of four (Introduction to Variables, Confounding and Intervening Variables, Research Design, and Introduction to Inference), with each treatment group having access to a different set of modules. Table 1 shows a complete description of access to modules by treatment group.

**Table 1:** Access to modules by treatment group. All groups had access to the Introduction and Research Questions, Theories, and Hypotheses modules. Each group was then randomly given access to two of the four remaining modules.

	Treatment Group					
Module	1	2	3	4	5	6
Introduction	x	x	x	x	x	x
Research Questions, Theories, and Hypotheses	x	x	x	x	x	x
Introduction to Variables	x	x	x			
Confounding and Intervening Variables	x			x	x	
Research Design		x		x		x
Introduction to Inference			x		x	x

Students were provided with access to their respective Canvas pages during the first week of the course and were notified about materials both via email and in class. Students were also aware that the effectiveness of the resource was being tested, but not aware of design details, including their treatment group. Students were sent invitations to their Canvas page multiple times in the weeks leading up to the midterm exam. It is important to note that students were encouraged, but not required, to use the supplementary materials, and no additional incentives (e.g., extra credit points) were awarded for their use. Moreover, because we controlled access to the Canvas pages, students could not access the Canvas pages for other treatment groups.

A particular strength of our design is that it was double-blind. Neither the students nor the instructors (professor and TAs) were aware of the treatment assignment of each student. We also did not provide instructors access to the Canvas pages or OSI modules to prevent them from adjusting their teaching given the contents of the OSI modules.

Five weeks into the 10-week course, students took a midterm exam worth 20% of the course grade. The midterm exam (included in S1 File) consisted of 18 questions, 13 of which are the focus of our impact evaluation. Four questions (1a-1d) asked about course content that was not covered by the supplemental resources, and one (2a) asked about content taught in the “Research Questions, Theories, and Hypotheses” OSI module, which was available to all six treatment groups. Consequently, these questions were dropped from our study. By limiting ourselves to this subset of 13 questions, we limit our analysis to questions that focused on content covered in the OSI materials and that were available to some treatment groups, but not others. S1 File presents descriptive statistics about student performance on exam questions. Table 2 shows a complete description of how modules and questions overlap. Question 3a asks about information covered by two modules, which should bias our estimates towards zero.

**Table 2:** Exam questions and OSI modules. Mapping between material covered in each module and material covered in each exam question.

	Q2			Q3		Q4				Q5			
Module	b	c	d	a	b	a	b	c	d	a	b	c	d
Introduction													
Research Questions, Theories, and Hypotheses													
Introduction to Variables	x	x	x										
Confounding and Intervening Variables				x	x								
Research Design				x						x	x	x	x
Introduction to Inference						x	x	x	x				

The unit of observation in this study is student-question. Treated units include those questions answered by students who had access to a relevant OSI module. Untreated units include student answers to questions covered by OSI material but *not* available to them. The main advantage of this research design is that errors associated with differences in skill and effort between students are reduced—each student acts as their own control group.

In total, there were 216 students enrolled in the course during the first week of the quarter. Of those, 189 students took the midterm exam. Forty-six students were dropped from the study either because they opted out or were minors at time of consent. Thus, our study includes 143 students and our student-question data set contains 1,859 observations (143 students × 13 questions). Treatment groups ranged in size from 18 to 28. We chose to evaluate the impact of the supplementary material on midterm exam performance so that students could have full access to the modules for the remainder of the quarter after they took the exam.

The exams were graded by five Political Science PhD students using the Gradescope application, with each PhD student grading the same set of questions across all students. To ensure consistency, we created a detailed rubric for each question and trained each grader on the grading rubric. Questions 3a and 3b were graded by one of the co-authors of this study, and all remaining questions were graded by PhD students serving as TAs for POLI 30, none of whom were directly involved in the study. TAs were not aware of treatment assignments when grading. TAs also had no access to the online modules, which eliminates the concern that they would modify their teaching to fit the material covered by the modules. As a robustness check, we conducted our impact evaluation with a subsample that excludes questions 3a and 3b to account for potential sources of bias. Results of this robustness check, shown in S1 File, are consistent with the main results.

Several strengths of the research design are worth highlighting. First, designing the midterm allows us to measure the effects on specific learning outcomes, rather than effects on overall course or exam grades, enabling us to provide direct evidence that the modules affect the specific outcomes for which they were intended. Second, the within-subject design offers various strengths that have been highlighted by the literature [43, 44]. It provides a more ethical way of testing the effects of OSI than alternative experimental designs since it ensures that all students receive some randomly determined subset of modules. That is, it was not the case that some students were given access while others were not. Additionally, the within-subject design increases the number of observations since each student is observed multiple times, increasing statistical power. It also allows for the inclusion of student fixed effects, which absorb student-specific variance. If student performance *across outcomes* looks very different from one student to another, then this fixed effect becomes a good predictor of the outcome and leads to more precisely estimated estimates. Finally, the within-subject design allows us to better detect student-level heterogeneity across subgroups of students since the individual treatment effects can be estimated for each student.

### OSI availability

Our experiment was implemented as an encouragement design where the assignment to treatment was randomized but treatment compliance was not obligatory. As a result, non-compliance (students having access to but not using the OSI modules) is present, meaning we cannot estimate the average treatment effect (ATE). We therefore first estimate the intent-to-treat effect (ITT); that is, the effect of having access to the instructional materials on exam question scores. We use OLS linear regression to estimate the ITT.

We estimate the regression using question-student level data. The regression model is:

$$\begin{array}{c} Y_{iq}=\beta TreatmentAssignment_{iq}+\gamma_{i}+\lambda_{q}+\epsilon_{iq} \end{array}$$ (1)

where *q* denotes each question and *i* denotes each student. *β* is the causal coefficient of interest. *Y*_*iq*_ denotes student performance on each exam question, which we measure using both percentages (0%-100%) and standardized scores. The treatment is a dummy variable indicating whether student *i* had access to supplemental modules addressing question *q*. *γ*_*i*_ are student fixed effects, and λ_*q*_ are question fixed effects. The exam question fixed effects should absorb any differences in grading across exam questions as well as factors that affect each question equally across students. For models that measure the dependent variable as standardized scores we do not include question fixed effects. Additional models with student-specific controls are included in S1 File and are consistent with the main results.

Student fixed effects control for any time-invariant observable or unobservable student-specific characteristic. The within-subject design means that each student acts as their own control group, allowing us to estimate within-subject variation in outcomes when they received treatment versus when they did not. Finally, because our treatment is assigned at the student level, we cluster the standard errors at the student level for all models. As a robustness check, we conduct randomization inference (shown in S1 File) and find that our main estimate is larger than 99.68% (*p* − *value* = 0.0032) of the 5,000 placebo estimates.

One concern may be spillover effects—students accessing OSI modules they were not assigned—which would violate identification assumptions and bias the estimates. We believe this is unlikely because students could not access OSI modules they were not assigned. Still, students may have shared their modules with other students. This also seems improbable because students would have needed either to share their confidential university username and passwords, or physically show the modules on their computers to other students. Because the experiment was conducted during the Covid-19 pandemic when courses were virtual, university facilities were closed, and many students were not living on campus, this seems unlikely. Even if spillover was present, this would mean students may have improved their scores for control questions, which would bias our estimates towards zero. In other words, even if spillover effects were present, they would bias estimates against finding treatment effects.

### OSI compliance

The ITT does not tell us the effect of students using the OSI resource on student learning, which is of great interest. By hosting the OSI modules on Canvas, we were able to collect information on treatment compliance—students engaging with the modules they were assigned. Yet, simply regressing module use on student outcomes would result in biased results because compliance is likely endogenous. Indeed, we find suggestive evidence of this: there is a positive correlation between pre-treatment GPA and engagement with the resource and suggestive evidence that underrepresented students engaged more with the resource, though neither of these differences are statistically significant.

Nevertheless, information on compliance does enable us to estimate the causal effect of the OSI resource on compliers, or the local average treatment effect (LATE) for compliers, also called the complier average causal effect (CACE), which estimates the average treatment effect (ATE) for compliers [45, Ch. 5]. In this study, the LATE is especially useful because it estimates the effect of the OSI resource on students who engaged with the resource.

We measure compliance in two ways (see Table 3 for descriptive statistics). First, we measure compliance by whether students viewed at least one OSI module at least once. This is a conservative measure that simply identifies whether a student was exposed to the treatment by looking at the modules available to them. Of the 182 students in the dataset, 122 students viewed at least one page. Second, we measure compliance by whether a student completed at least one quiz. Of the 182 students, 93 completed at least one module quiz. This measure aligns more with our conceptual understanding of the treatment: actual engagement with the OSI modules. However, we interpret this second measure with caution as it categorizes students that used the OSI modules without answering quiz questions as noncompliers.

**Table 3:** Summary statistics. Student-level summary statistics: modules use, GPA, exam question scores, and underepresented student status.

Variable	N	Mean	Median	Std. Dev.	Min	Max
Page views	143	29.25	24.00	27.93	0.00	141.00
Quiz completions	143	2.64	3.00	2.90	0.00	8.00
Incoming GPA	141	3.48	3.61	0.50	1.10	4.00
Exam Question Score	143	78.10	80.77	13.26	29.21	98.97
Variable	N	Perc.				
Viewed ≥ one page	143					
Yes	117	81.8%				
No	26	18.2%				
Completed ≥ one quiz	143					
Yes	73	51%				
No	70	49%				
URM Status	131					
Not URM	92	70.2%				
URM	39	29.8%				

Since compliance is endogenous to student-specific characteristics, we estimate the LATE with an instrumental variables (IV) design [45, 157-160] where we regress the outcome on the treatment compliance, using treatment assignment as an instrument for treatment compliance. We use the two-stage least squares (2SLS) to estimate the LATE. The first stage is:

$$\begin{array}{c} Treated_{iq}=\beta_{1}TreatmentAssignment_{iq}+\gamma_{i}+\lambda_{q}+\epsilon_{iq} \end{array}$$ (2)

where *TreatmentAssignment*_*iq*_ denotes whether student *i* had access to online supplemental modules addressing question *q*. *Treated*_*iq*_ denotes whether student *i* used online supplemental modules addressing question *q*. Results for the first stage show that the treatment assignment is a valid and strong instrument for whether students used the modules. We report the results of the first stage in S1 File.

In the second stage, we regress the outcome on the fitted values from the first stage:

$$\begin{array}{c} Y_{iq}=\beta_{2}{\hat{Treated}}_{iq}+\gamma_{i}+\lambda_{q}+e_{iq} \end{array}$$ (3)

The coefficient of interest is *Y*_*iq*_, which estimates the LATE. The reduced form for this instrumental variable is the ITT analysis from the previous section.

### Heterogeneous effects

To explore heterogeneous effects, also known as treatment-by-covariate effects [45] or conditional average treatment effects (CATE), we obtained de-identified student information on cumulative GPA prior to taking POLI 30 and minority student status. While we pre-registered the model specification presented here, we did not pre-register the particular student-specific covariates to be analyzed as there was uncertainty about which student-specific information would be made available to us by UCSD. The student-specific information we received from UCSD was anonymized so that we could not identify individual participants. The results should therefore be considered exploratory. Table 3 shows descriptive statistics for these variables.

We estimate heterogeneous effects using linear regression:

$$\begin{array}{c} Y_{iq}=\delta TreatmentAssignment_{iq}\times StudentChar_{i}+\gamma_{i}+\lambda_{q}+\epsilon_{iq} \end{array}$$ (4)

where *StudentChar*_*i*_ denotes the student-specific characteristic for which we have data (underrepresented or pre-treatment cumulative GPA). The interaction between this variable and the treatment assignment allows us to estimate the heterogeneous effects of interest, making *δ* the main coefficient of interest.

## Results

**Fig 2:**
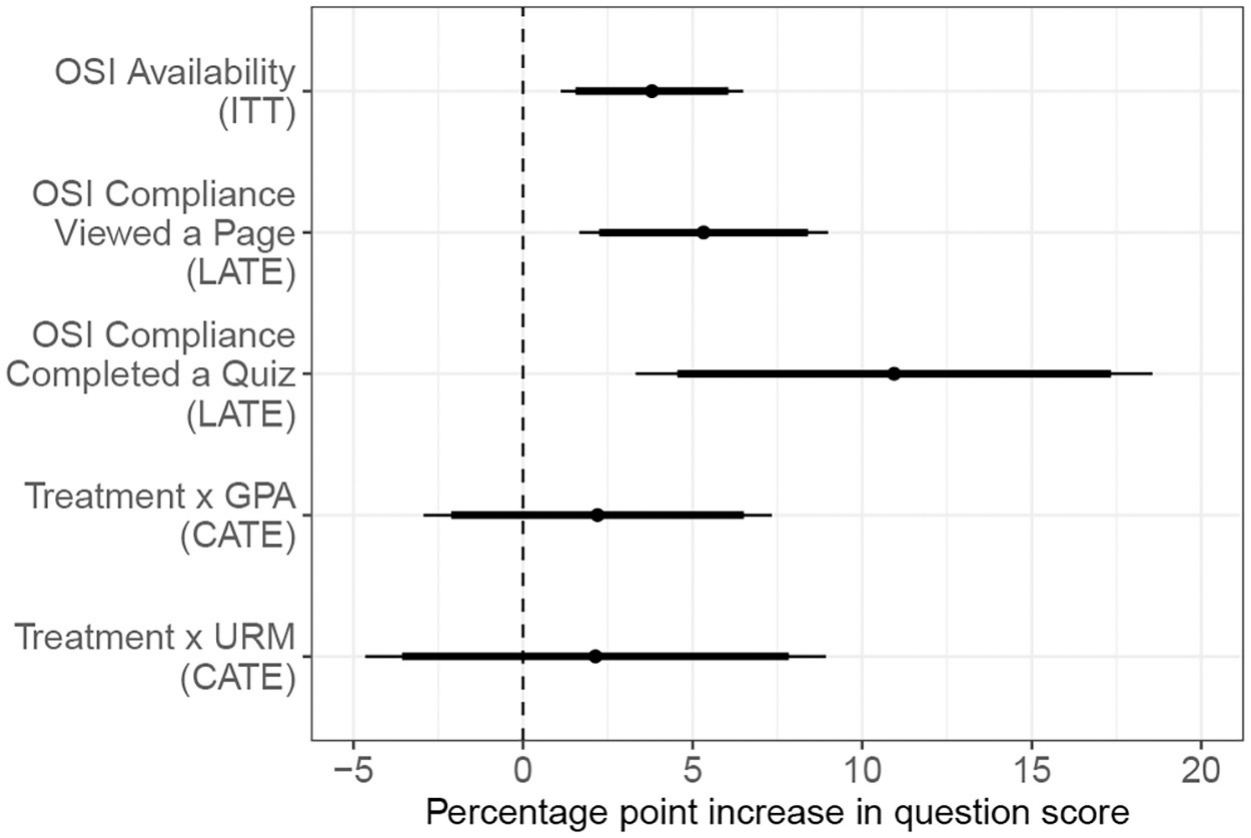
Coefficient plot of main results. Plot shows point estimates from the main results using exam question scores in percent as the outcome measure with 90% and 95% confidence intervals represented with thick and thin lines, respectively. From top to bottom, plot shows effect of OSI availability (ITT) and OSI use (LATE) measured as viewing a page and completing a quiz, respectively, and effects of OSI availability conditional on student GPA and underrepresented status (URM) on exam question scores. Analysis includes student and question fixed effects. Robust standard errors are clustered at the student level. ITT = Intent to treat. LATE = Local average treatment effect. URM = student underrepresented status.

We find that access to the OSI modules significantly increased student performance. Fig 2 visualizes the main results using exam question scores in percent as the outcome variable. On average, having access to the modules increased question scores by 3.8 percentage points, which translates to an increase of 0.14 standard deviations. While these estimates may appear small, they translate to about one third of a letter grade increase in the overall exam grade. That is, the average effects suggest that if students had access to the treatment for all four modules, their exam grades would have increased by 3 points out of 100. Further, point estimates from the LATE analysis of compliers suggest that questions scores were 5.3 points higher when students viewed at least one page related to that question, and nearly 11 percentage points higher when students completed at least one quiz. Finally, we do not find evidence that treatment effects varied across students depending on their pre-treatment cumulative GPA or underrepresented status.

Table 4 shows the full regression output of the main results for both ITT and LATE estimations using both measures of the outcome variable. Models 1 and 4 show the results for OSI availability (ITT), while Models 2, 3, 5, and 6 show the second stage results for OSI compliance (LATE) using both measures of compliance (viewing a page and completing a quiz). For the IV-2SLS models, we estimate the first-stage cluster-robust *F* statistics and find that they are above conventional levels for a strong instrument, meaning that the treatment assignment is a valid instrument for both measurements of compliance (*F* = 425.8 for page views and *F* = 116.3 for quiz completion). The instrument being randomly assigned as part of an experimental design further validates its use. We report results for the first stage in S1 File.

**Table 4:** Main results. Effects of OSI on student grades in exam questions. Models 1–3 use question grades in percent as outcome measure. Models 4–6 use standardized question grade as outcome measure. Models 1 and 4 estimate the ITT, models 2 and 4 estimate the LATE using module view as compliance, models 3 and 6 estimate the LATE using quiz taking as compliance.

	Question Score					
	Percent			Standardized		
	(1)	(2)	(3)	(4)	(5)	(6)
OSI Available	3.801***			0.143***		
	(1.371)			(0.050)		
OSI Compliance—viewed a page (instrumented)		5.325***			0.198***	
		(1.870)			(0.069)	
OSI Compliance—completed a quiz (instrumented)			10.943***			0.418***
			(3.886)			(0.146)
Model	OLS	IV-2SLS	IV-2SLS	OLS	IV-2SLS	IV-2SLS
Student FE	Yes	Yes	Yes	Yes	Yes	Yes
Question FE	Yes	Yes	Yes	No	No	No
Observations	1,859	1,859	1,859	1,859	1,859	1,859
R^2^	0.466	0.465	0.461	0.257	0.255	0.250

*Note:* Standard errors clustered by student in all models.

*p<0.1;

**p<0.05;

***p<0.01.

As expected, the LATE estimates are larger than the ITT estimates, and LATE estimates for compliance measured as quiz-taking are larger than those of compliance measured as exposure to the treatment. Fig 2 and Table 4 show that giving students access to OSI resources improved question scores (Models 1 and 4), and the effect was larger for students who viewed the resource (Models 2 and 5), and even larger for students who engaged with it (Models 3 and 6). This suggests that the OSI modules help students more the more they engage with them.

Table 5 shows full regression output for heterogenous effects. We find that the direction of both interaction estimates are positive, perhaps suggesting that students with higher GPAs and underrepresented students benefited more from the modules. However, the estimates are not statistically significant at any conventional level. We therefore find no evidence that the modules had differential impacts on students depending on their GPAs or underrepresented status.

**Table 5:** Heterogeneous treatment effects. Treatment-by-covariate effects of OSI access on exam question grades given students’ incoming cumulative GPA and underrepresented minority status. Models 1 and 2 use question grade in percent as outcome. Models 3 and 4 use standardized question grade as outcome.

	Question Score			
	Percent		Standardized	
	(1)	(2)	(3)	(4)
OSI Available	−3.861	3.343**	−0.177	0.134**
	(9.543)	(1.570)	(0.353)	(0.059)
OSI Available × GPA	2.197		0.092	
	(2.620)		(0.097)	
OSI Available × URM		2.136		0.059
		(3.464)		(0.130)
Student FE	Yes	Yes	Yes	Yes
Question FE	Yes	Yes	No	No
Observations	1,833	1,703	1,833	1,703
R^2^	0.469	0.465	0.258	0.255

*Note:* Standard errors clustered by student in all models.

*p<0.1;

**p<0.05;

***p<0.01.

### Survey results

In addition to encouraging empirical results, we received positive feedback from students. We conducted a survey at the end of the POLI 30 course to elicit student views on the course and the supplemental modules. While the sample is relatively small, it suggests that the modules were well-received.

Of those who answered the survey, 17 out of 19 respondents reported using the resources. All 17 of these students reported that the resources were helpful (Yes/No question, “Did you find these resources helpful?”) Further, on a scale of 0–5, where 0 indicates “strongly disagree” and 5 indicates “strongly agree,” students generally agreed that the resources helped them get a better grade (mean = 4, range = 3–5); that they would recommend these resources to future POLI 30 students (mean = 4.53; range = 3–5), and that they would like to continue having access to the materials (mean = 4.63; range = 3–5).

## Discussion

This study presents a successful effort to improve undergraduate data literacy education by complementing a data course with asynchronous OSI. Using a pre-registered experimental design, we find that OSI resources significantly improve student learning, and that all students benefit equally regardless of their prior academic performance or minority student status. Moreover, point estimates suggest that the benefits for student learning are greater the more deeply students engage with the OSI resources.

Our findings carry important implications for instruction in data literacy in higher education. As many colleges and universities struggle to support the development of students’ data literacy skills in increasingly resource constrained environments, this study provides compelling evidence that asynchronous OSI can provide a time- and cost-efficient means of improving student learning in this critical area.

An important caveat is that the study was implemented during the Covid-19 pandemic when classes were held entirely remotely. Future research could study the effect of OSI resources on student learning when classes are held in-person, as engagement may differ in important ways. For example, students might have gained more from the resources if they had the opportunity to sit together to work on quizzes and discuss instructional videos. On the other hand, remote instruction may have made it more natural for students to learn from online modules, given that online instruction was the default setting for the class. Furthermore, while we provide evidence that these resources strengthen data literacy skills in an introductory course, further research is needed to examine their downstream effects, for example, on performance in subsequent advanced courses, GPA, graduation, and labor market outcomes.

To conclude, given the importance of data literacy and the difficulties students face, we present evidence that OSI resources such as ours can play an important role in supporting student learning and closing achievement gaps. They may also benefit faculty and TAs who teach these courses, especially those in resource-constrained environments often shaped by high student-to-TA and faculty ratios. We hope these materials are widely shared, replicated, or used to inspire new projects to support student learning in this critical area of higher learning.

## Supporting information

S1 File Supporting information. (PDF)
