# Supplementary material for: Supplemental online resources improve data literacy education: Evidence from a social science methods course
Source: PLoS One. 2024 Dec 19;19(12):e0315318. doi: 10.1371/journal.pone.0315318 (PMC11658515; doi:10.1371/journal.pone.0315318)
Supplement: S1 File — (PDF) [file pone.0315318.s001.pdf]

Supporting Information
Supplemental Online Resources Improve Data Literacy Education

Marco Alcocer[a,c], Leonardo Falabella[a,b], Alexandra Lange[a], Nicholas Smith[a], and Maureen Feeley[a]

[a]University of California, San Diego
[b]The London School of Economics and Political Science
[c]University of California, Merced

Contents

S1 Modules and Module Content 2

S2 Measuring Student Learning: Midterm Exam 4

S3 Research Design 7

S4 Randomization Inference 8

S5 OSI Compliance: First Stage 10

S6 Module Use by Students 11

S7 Student Performance on Exam Questions 13

S8 Main Results Robustness Checks 14

S1 Modules and Module Content

Table S1: **Modules and module content.**

Module	Content
Introduction	1. Welcome to Foundations of Quantitative Research in Political Science<br>2. Video: Introduction
Research Questions, Theories, and Hypotheses	1. Introduction to Research Questions, Theories, and Hypotheses<br>2. Video: Research Questions, Theories, and Hypotheses<br>3. Quick Recap: Research Questions, Theories, and Hypotheses<br>4. Knowledge Check: Research Questions, Theories, and Hypotheses<br>5. Reflection: Research Questions, Theories, and Hypotheses
Introduction to Variables	1. Introduction to Variables<br>2. Variables and Values<br>3. Types of Variables<br>4. Dependent and Independent Variables<br>5. The Unit of Analysis<br>6. Video: Measuring Variables<br>7. Quick Recap: Introduction to Variables<br>8. Knowledge Check: Introduction to Variables<br>9. Reflection: Introduction to Variables
Confounding and Intervening Variables	1. Introduction to Confounding and Intervening Variables<br>2. Video: Criteria of Confounding Variables<br>3. Quick Recap: Confounding Variables<br>4. Knowledge Check: Confounding Variables<br>5. Video: Confounding vs. Intervening Variables<br>6. Quick Recap: Intervening Variables<br>7. Knowledge Check: Confounding vs. Intervening Variables<br>9. Reflection: Confounding and Intervening Variables
Research Design	1. Introduction to Research Design<br>2. Video: Experiments as the Gold Standard<br>3. Quick Recap: Experiments<br>4. Video: Observational Research Design<br>5. Quick Recap: Observational Research Design<br>6. Natural Experiments and Quasi-Experiments<br>7. Knowledge Check: RCTs, Natural Experiments, Quasi-Experiments, Observational Studies<br>9. Reflection: Research Design

Table S1 (*Continued*): **Modules and module content.**

**Module**	**Content**
Introduction to Inference	1. Introduction to Inference<br>2. Video: Populations and Samples<br>3. Population Parameters and Sample Statistics<br>4. Types of Samples<br>5. Quick Recap: Populations and Samples<br>6. Video: Margin of Error<br>7. Quick Recap: Margin of Error<br>8. Knowledge Check: Population and Samples, Margin of Error<br>9. Video: Random Sample vs Random Assignment<br>10. Quick Recap: Random Sample vs Random Assignment<br>11. Reflection: Introduction to Inference
Hypothesis Testing	1. Introduction to Hypothesis Testing<br>2. Video: Introduction to Bivariate Hypothesis Testing<br>3. Quick Recap: Hypothesis Testing<br>4. Selecting the Appropriate Hypothesis Test<br>5. Knowledge Check: Hypothesis Testing<br>6. Reflection: Hypothesis Testing
Regression Analysis	1. Regression Analysis: Introduction<br>2. Video: Regression Analysis Review<br>3. Video: From the Data to the Regression Equation<br>4. Video: Regression and Hypothesis Testing<br>5. The R-squared<br>6. Multivariate Regression: Introduction<br>7. Video: Regression and Confounding Variables<br>8. Standard Error<br>9. Video: Reading Regression Tables<br>10. Knowledge Check: Regression Analysis<br>11. Reflection: Regression Analysis
Working with Data	1. Intro: Datasets and Working with Data<br>2. Video: Working with Datasets<br>3. Knowledge Check: Datasets<br>4. Quick Recap: Working with Data<br>5. Reflection: Working with Data
Visualizing Data	1. Intro to Visualizing Data<br>2. How to Make Charts and Visualizations<br>3. Knowledge Check: Charts and Visualizations<br>4. Video: Questioning Data Visualization<br>5. Knowledge Check: Questioning Data Visualizations<br>4. Video: Questioning Data Visualization<br>5. Quick Recap: Visualizing Data<br>6. Reflection: Visualizing Data

S2 Measuring Student Learning: Midterm Exam

We designed a midterm exam to evaluate the impact of the OSI modules on student learning. We wrote the midterm exam so that each question asked about a specific concept or skill that was addressed *both* in lectures (every student was exposed to this) and in the OSI modules (students were only exposed to a subset of modules). This design ensured that students were answering questions about material presented in class, meaning that no student was unfairly disadvantaged, and some of these questions were supplemented by the OSI modules students were assigned.

The midterm exam consists of 18 questions, 13 of which are the focus of our impact evaluation. Four questions (1a-1d) asked about course content that was not covered by the supplemental resources, and one (2a) asked about content taught in the "Research Questions, Theories, and Hypotheses" OSI module, which was available to all six treatment groups. Consequently, these questions were dropped from our study. By limiting ourselves to this subset of 13 questions, we limit our analysis to questions that focused on content covered in the OSI materials that were available to some treatment groups and not others. The midterm exam is provided below.

1. The star of the UCSD basketball team has played well lately. In the last five games, she has point totals of:

   22, 30, 18, 16, 24

   Answer the following questions. Show your work in order to get full credit.

   a. What type of variable is "point total," as described above: nominal, ordinal, or interval/continuous? Why?

   b. What is the median point total (as a number)?

   c. What is the mean point total (as a number)?

   d. What is the variance of this sample of point totals (as a number)?

2. Consider the following hypothesis: "Candidates tend to spend more money in close elections."

    a. What is the dependent variable? What is the independent variable?

    b. What is the unit of analysis?

    c. How would you measure the closeness of elections? Justify why you believe this is a good measure.

    d. What type of variable would your measurement strategy produce (e.g. ordinal, nominal, interval, or ratio?). Explain why, using the definition of this type of variable.

3. Consider the following hypothesis: "Famines are less likely to happen in countries where there is press freedom."

    a. Can you think of a confounding variable in this hypothesis? Please justify your answer. In your justification, be sure to demonstrate that you understand the concept of confounding variables by identifying the criteria of confounding variables and explaining how your confounding variable meets each criterion.

    b. Can you think of an intervening variable in this hypothesis? Please justify your answer. In your justification, be sure to demonstrate that you understand the concept of intervening variables by identifying the criteria of intervening variables and explaining how your intervening variable meets each criterion.

4. Suppose we want to study whether UCSD students approve of the cost of parking on campus. We set up a table by the entrance of two parking structures on campus and ask students who are walking in what they think about the cost of parking.

    a. What is the population in this study?

b. Is this a probability or a non-probability sample? Please justify your answer. In your justification, be sure to demonstrate that you know the difference between probability and non-probability samples.

c. When can we make inferences about populations from samples?

d. Could we make inferences about UCSD student approval of parking costs using the sampling procedure described in the prompt? Why or why not?

5. Suppose you want to answer the following research question: "Do people become more receptive to being vaccinated if they watch a scientist explain how vaccines work?"

   a. Describe an experiment that would enable us to answer this research question (3-4 sentences).

   b. In this experiment, what would be the treatment?

   c. What would be the treatment group? And the control group?

   d. How would this experiment mitigate our concerns about confounds?

S3 Research Design

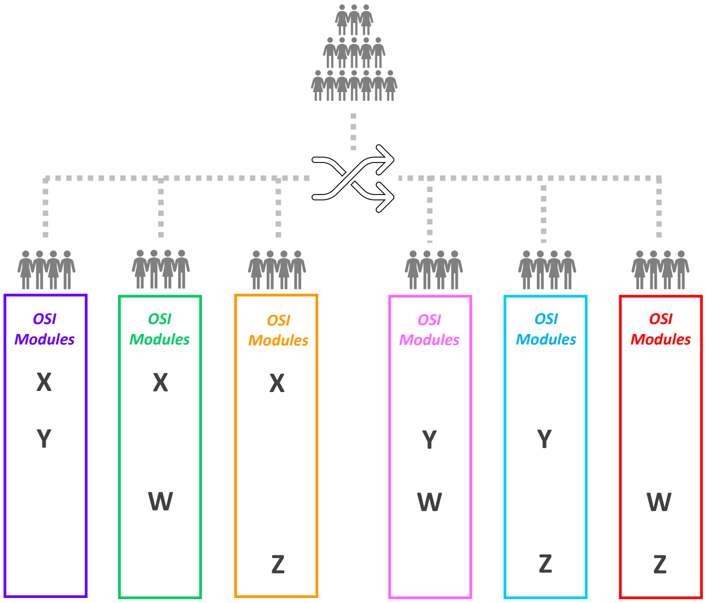

Figure S1: Students were randomly assigned into six treatment groups. Each treatment group had access to a different subset of two out of four modules.

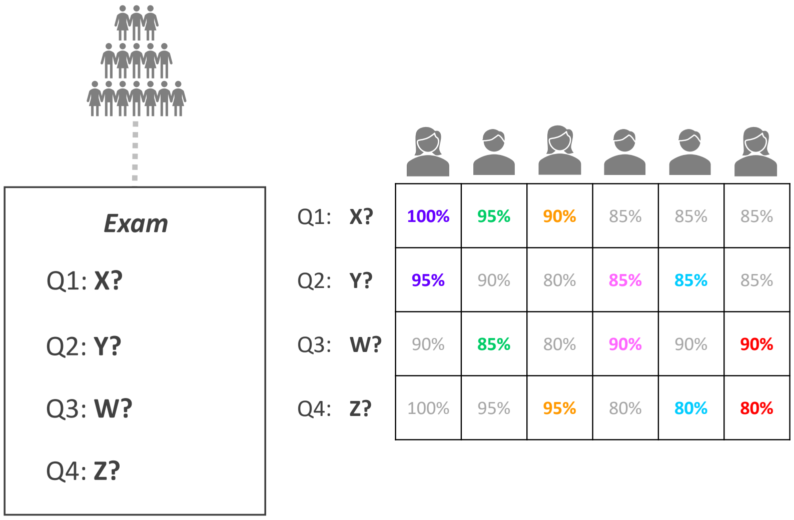

Figure S2: Each time a student answered a question, there was a random chance that the question content was taught in modules that the student could access.

S4 Randomization Inference

One possible concern is that the results we observe are due to the specific groups created by randomization. That is, it may be that, simply by chance, the treated group was different than the control group in some dimension that explains all or part of the results. Because our study is a within-unit design, this concern is minimal since each student acts as their own control. Nevertheless, we assess how unusual our results are when compared to other possible random assignments by conducting randomization inference [1]. Specifically, we simulate 5,000 alternative random assignments following the original treatment assignment method (randomly assigning each student to one of six treatment groups). For each of the 5,000 alternative treatment assignments, we then estimate the effect of the treatment on student learning using the main regression model, specified below. Finally, we use these 5,000 regression coefficients to see how (un)likely it is that we observe our main results just by chance.

$$Y_{iq} = \beta Placebo_{iq} + \gamma_i + \lambda_q + \epsilon_{iq} \quad (1)$$

where q denotes each question and i denotes each student. β is the causal coefficient of interest. Y_{iq} denotes student performance in each exam question, which we measure using both percentages (0%-100%) and standardized scores. The treatment is a dummy variable indicating whether student i had access to supplemental modules addressing question q. γ_i are student fixed effects, and λ_q are question fixed effects. The exam question fixed effects should absorb any differences in grading across exam questions as well as factors that affect each question equally across students.

Figure S3 contains the distribution of all 5,000 coefficients. The vertical line displays the observed coefficient in our original treatment assignment, thus indicating that our observed coefficient is highly unusual against other possible random assignments (i.e., placebo statuses). Specifically, our observed coefficient ($\hat{\beta} = 3.8$) is higher than 99.68% of all placebo statuses (p-value = 0.0032). We can thus confidently reject the sharp null that our treatment

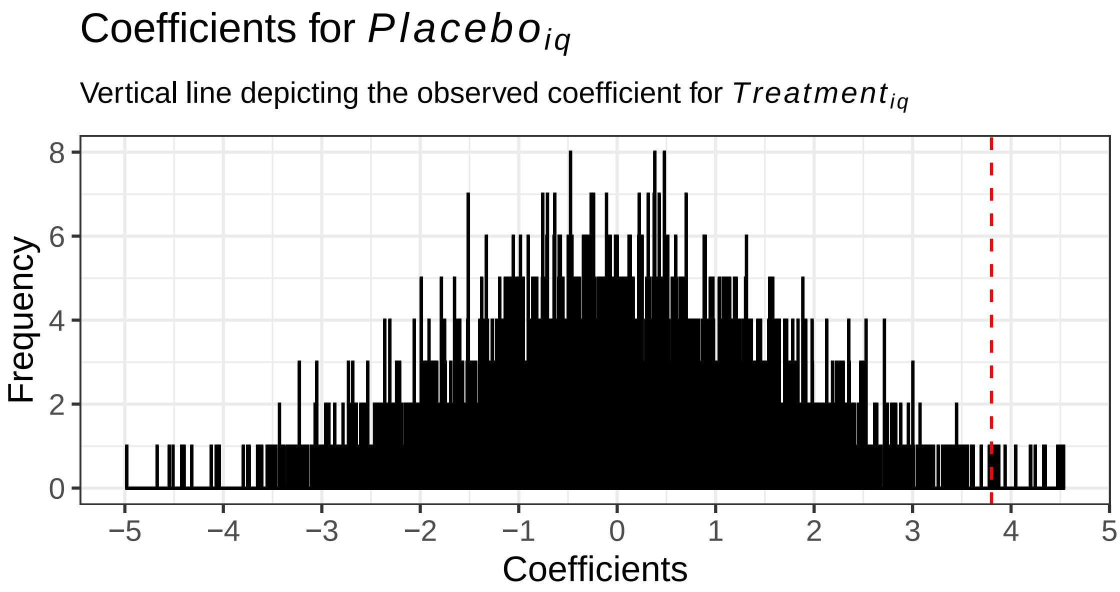

Figure S3: Randomization Inference. The histogram reports the coefficients of 5,000 simulated regression with placebo statuses generated according to the original treatment assignment method. The p-value for the sharp null hypothesis test is 0.0032, indicating that the observed coefficient in our original result ($\hat{\beta} = 3.8$, depicted by the vertical line) is significantly unusual against other possible random assignments.

had no effect on any student.

S5 OSI Compliance: First Stage

Table S2 reports the first stage regressions for the main LATE, or CACE, results. Results show that viewing a page and completing a quiz are both strong and valid instrumentals in our experimental setting, with cluster-robust IV F statistics ranging from 98.8 to 453.6.

Table S2: Compliance to treatment, first stage.

	Compliance			
	Viewed a page		Completed a quiz	
	(1)	(2)	(3)	(4)
Treatment (OSI available)	0.714***	0.723***	0.347***	0.342***
	(0.036)	(0.035)	(0.034)	(0.036)
Cluster-robust IV F Stat	425.7918	453.6375	116.3489	98.8259
Student FE	Yes	Yes	Yes	Yes
Question FE	Yes	No	Yes	No
Observations	1,859	1,859	1,859	1,859
R^2	0.755	0.748	0.538	0.448

Note: *p<0.1; **p<0.05; ***p<0.01
Standard errors clustered by student in all columns.

S6 Module Use by Students

Figure S4 and Table S3 report descriptive statistics on module use, showing that students were generally motivated to view OSI pages but not as willing to answer quiz questions. Table S3 also shows that approximately 30% of participants are underrepresented minority students.

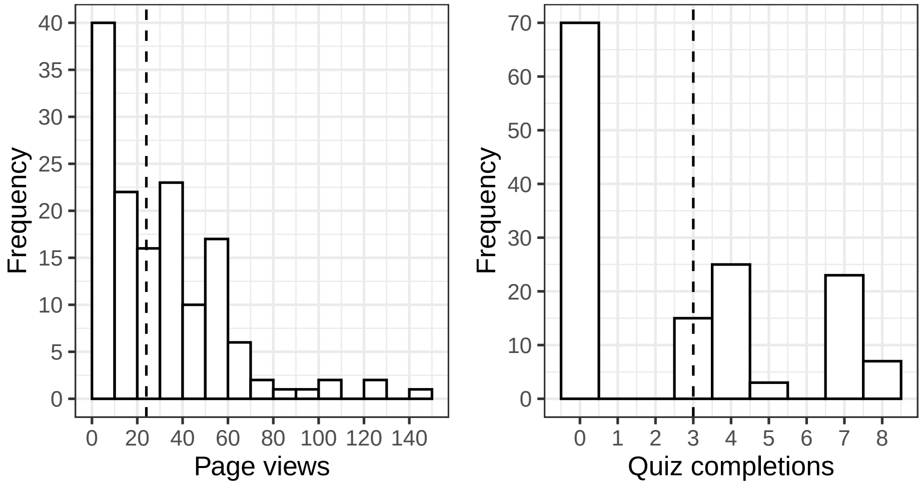

Figure S4: Module use distribution. Vertical lines depicting the median values.

Table S3: Student-level summary statistics: percentages of students who viewed at least one page, took at least one quiz, and by URM status. While more than 80% of students viewed at least one OSI page, almost half (49%) did not take any quiz.

Variable	N	Percent
Viewed at least one page	143	
... Yes	117	81.8%
... No	26	18.2%
Completed at least one quiz	143	
... Yes	73	51%
... No	70	49%
URM Status	131	
... Not URM	92	70.2%
... URM	39	29.8%

S7 Student Performance on Exam Questions

Table S4 describes the performance of students across the exam questions included in our analysis (2b-5d). We report exam scores measured as percentages, which does not reflect the weights of each question in the exam. Students struggled to explain when we can make inferences about populations from samples (4c) and how randomized experiments can mitigate concerns about confounds (5d), and did well in questions that asked to identify the unit of analysis in a hypothesis (2b), identify the type of a variable (2d), and identify the treatment and control groups in an experimental setting.

Table S4: Summary statistics for question scores. The most challenging question (5d) asked students to explain how a randomized experiment mitigates concerns about confounds. The least challenging question (5c) asked students to identify the treatment and control groups in an experimental setting.

Question	N	Mean	Median	Std. Dev.	Min	Pctl. 25	Pctl. 75	Max
q2b	143	95.31	100.00	13.10	0.00	100.00	100.00	100
q2c	143	83.71	90.00	15.41	40.00	70.00	100.00	100
q2d	143	93.78	100.00	14.08	0.00	90.00	100.00	100
q3a	143	80.87	90.00	25.54	0.00	75.00	100.00	100
q3b	143	83.39	100.00	28.90	0.00	75.00	100.00	100
q4a	143	87.88	100.00	25.80	33.33	100.00	100.00	100
q4b	143	82.42	100.00	27.79	0.00	71.43	100.00	100
q4c	143	50.63	60.00	29.05	0.00	40.00	80.00	100
q4d	143	63.64	60.00	34.12	0.00	40.00	100.00	100
q5a	143	67.55	73.33	24.38	0.00	53.33	86.67	100
q5b	143	89.37	100.00	28.36	0.00	100.00	100.00	100
q5c	143	95.45	100.00	19.60	0.00	100.00	100.00	100
q5d	143	41.26	50.00	42.51	0.00	0.00	100.00	100

S8 Main Results Robustness Checks

Table S5 reports the results of a robustness check. Whereas most questions were graded by TAs without access to the OSI modules, questions 3a and 3b where graded by one of the co-authors of the OSI modules. Even though the co-author/grader did not had access to the treatment statuses of students, they could not see students' names while grading because student names were omitted in the grading process. However, we decided to run our OLS regression with a subset of the data that excludes questions 3a and 3b because of the potential for implicit bias. The results of our experiment remain consistent after dropping questions 3a and 3b from the data.

Table S5: Robustness check: Main results with a subset excluding questions 3a and 3b (Confounding and Intervening Variables), which were graded by a co-author of the supplemental online resources. The co-author/grader had access to assignment to treatment by student, but could not see students' names while grading since student names were omitted by the Gradescope application. All remaining questions were graded by TAs without access to the resources.

	Question Score					
	Percent			Standardized		
	(1)	(2)	(3)	(4)	(5)	(6)
Treatment (OSI available)	3.310**			0.118*		
	(1.647)			(0.061)		
Compliance (viewed a page)		4.718**			0.165**	
		(2.248)			(0.083)	
Compliance (completed a quiz)			10.603**			0.378**
			(5.105)			(0.192)
Model	OLS	IV-2SLS	IV-2SLS	OLS	IV-2SLS	IV-2SLS
Student FE	Yes	Yes	Yes	Yes	Yes	Yes
Question FE	Yes	Yes	Yes	No	No	No
Observations	1,573	1,573	1,573	1,573	1,573	1,573
R^2	0.497	0.496	0.491	0.265	0.264	0.258
Note:						*p<0.1; **p<0.05; ***p<0.01<br>Standard errors clustered by student in all columns.

Finally, Table S6 shows the results of regression models specified in our pre-analysis plan (found here: https://osf.io/scx6r), but which we decided to omit from the body of

the paper. The PAP specified including a matrix with student-specific covariates in our regression models. Because the models included in the body of the paper include student fixed effects, student-invariant characteristics are controlled for, in a way that adding student-specific covariates was a redundancy that also amounted to a loss of data, since some students had missing values for these covariates. We chose to report the results without student-specific covariates in the body of the paper and the results with student-specific covariates in the Appendix. Results from the model with student-specific covariates are nearly identical, with the minor differences being due to the smaller number of observations.

Table S6: Robustness check: Main results including student-specific covariates (GPA and URM) and dropping observations with missing covariate values.

	Question Score					
	Percent			Standardized		
	(1)	(2)	(3)	(4)	(5)	(6)
Treatment (OSI available)	3.959***			0.152***		
	(1.475)			(0.055)		
Compliance (viewed a page)		5.601***			0.212***	
		(2.034)			(0.075)	
Compliance (completed a quiz)			11.438***			0.440***
			(4.224)			(0.159)
Model	OLS	IV-2SLS	IV-2SLS	OLS	IV-2SLS	IV-2SLS
Student Covariates	Yes	Yes	Yes	Yes	Yes	Yes
Student FE	Yes	Yes	Yes	Yes	Yes	Yes
Question FE	Yes	Yes	Yes	No	No	No
Observations	1,677	1,677	1,677	1,677	1,677	1,677
R^2	0.467	0.465	0.462	0.256	0.252	0.247

Note: *p<0.1; **p<0.05; ***p<0.01
Standard errors clustered by student in all columns.

References

[1] Gerber AS, Green DP. Field Experiments: Design, Analysis, and Interpretation. Illustrated edition ed. New York: W. W. Norton & Company; 2012.
